# Essential Nutrition and Food Systems Components for School Curricula: Views from Experts in Iran

**Published:** 2017-07

**Authors:** Sanaz SADEGHOLVAD, Heather YEATMAN, Nasrin OMIDVAR, Anne-Maree PARRISH, Anthony WORSLEY

**Affiliations:** 1. School of Health and Society, Faculty of Social Sciences, University of Wollongong, New South Wales, Australia; 2. Dept. of Community Nutrition, School of Nutrition Sciences and Food Technology, Shahid Beheshti University of Medical Sciences, Tehran, Iran; 3. Center for Physical Activity Research, School of Exercise and Nutrition Sciences, Deakin University, Burwood, Melbourne, Australia

**Keywords:** Nutrition, Food systems, Knowledge, Education, School, Iran

## Abstract

**Background:**

This study aimed to investigate food experts’ views on important nutrition and food systems knowledge issues for education purposes at schools in Iran.

**Methods:**

In 2012, semi-structured, face-to-face or telephone interviews were conducted with twenty-eight acknowledged Iranian experts in food and nutrition fields. Participants were selected from four major provinces in Iran (Tehran, Isfahan, Fars and Gilan). Open-ended interview questions were used to identify nutrition and food systems knowledge issues, which experts considered as important to be included in school education programs. Qualitative interviews were analyzed thematically using NVivo.

**Results:**

A framework of knowledge that would assist Iranian students and school-leavers to make informed decisions in food-related areas was developed, comprising five major clusters and several sub-clusters. Major knowledge clusters included nutrition basics; food production; every day food-related practices; prevalent nutritional health problems in Iran and improvement of students’ ethical attitudes in the food domain.

**Conclusion:**

These findings provide a guide to curriculum developers and policy makers to assess current education curricula in order to optimize students’ knowledge of nutrition and food systems.

## Introduction

The literature across several nations, including Europe (1), United States (2), and Iran (3) demonstrate the potential of schools to deliver important food and nutrition messages is yet to be reached. The importance of food and nutrition education is discussed in the literature (4), but to date, there is little published research identifying what students need to learn about nutrition and food systems more broadly.

Globally, different aspects of nutrition and food systems knowledge required for consumers to make informed food choices or to improve their dietary behaviors have been separately documented, covering a wide range of issues, such as food safety (5), use of food labels (6), cooking skills (7), and other food and nutrition topics.

Several assessment tools have been developed to measure particular sub-components of nutrition or food system knowledge such as dietary recommendations, food sources of nutrients, daily food selections, nutrition-related health problems (8), food system sectors, food sources, sustainability, local foods and hunger (9).

A major gap in the literature to date includes investigation of a broad range of important nutrition and food systems knowledge issues within one study. Consequently, there is little strategic guidance for food educators, education curriculum developers, and policy makers against which to assess and build comprehensive education programs. This research addresses this issue.

The “new nutrition science” (NNS) takes a broad view of nutrition and incorporates various food-related issues from different food and nutrition disciplines. The NNS reflects biological, social and environmental aspects of nutrition science that affect food systems and people’s food decision-making (10). However, this multidimensional perspective has been neglected to date in the food and nutrition knowledge literature.

Food and nutrition knowledge literature related to Iran has three major focuses. First, studies which assess a target population’s knowledge of a particular nutrition and food-related issues such as food labels (11), food groups (12), and food safety (13). Second, studies which raise the importance of nutrition education for particular health-related outcomes, for example preventing obesity (14), osteoporosis (15) and cardiovascular diseases (16). Third, studies that investigate aspects of nutrition knowledge associated with balanced, healthy eating habits for particular populations. For example, research directed attention to nutrition during pregnancy (17, adolescents’ knowledge of healthy dietary behavior (18), and knowledge for healthy eating among college athletes (19). However, there is not a study that identifies a broad range of nutrition and food systems issues essential for Iranians to know or a study that assesses Iranian’s knowledge of a wide range of food-related issues.

Knowledge, the focus of this investigation, is a complex concept (20). The incorporation of the epistemology of knowledge framework will assist in understanding the study findings. This study employed a holistic framework of knowledge developed by Yang (20) based on three facets of knowledge 1) explicit, 2) implicit and 3) emancipatory, reflecting the different processes of knowing in adult learning. The explicit facet represents scientific aspects; the implicit facet reflects practice, and the emancipatory facet is carried by values and ethics (20). This approach is consistent with the multidimensional nature of the NNS, whose dimensions of knowledge cover scientific (explicit) aspects of nutrition; food and nutrition-related skills (implicit); and cultural and values-based (emancipatory) factors.

## Methods

### Participants

Participants were drawn from various food-related fields to ensure the research incorporated expert views on the different dimensions of nutrition and food system issues consistent with the New Nutrition Science (NNS). A mix of Iranian experts was identified for recruitment, including four public health nutritionists, four nutritionists, four dietitians, four food scientists, two environmental scientists, two veterinary physicians, two agriculture scientists and four high school teachers (health teachers and home economics teachers) across the country. Participants were recruited through purposive sampling and snowballing. Experts were considered eligible for inclusion if they were acknowledged academics in top-ranked universities, practitioners who had key roles in professional governmental and non-governmental organizations, and recognized experts in private practice. Experts were invited to participate in person or by telephone. The dates and venues for the interviews were then arranged and participants were given the interview questions, participant information sheet and consent form (all in Farsi) prior to the interviews.

Ethics approval was received from the Human Research Ethics Committee (Health and Medical) of the University of Wollongong. Anonymity was assured and participants’ identities were replaced with alphabetical letters and numbers on any potentially identifying materials such as notes, transcripts, and recordings.

### Data Collection

Open-ended interview questions were developed by the study authors and reviewed by an academic review panel. Questions explored the experts’ attitudes regarding important nutrition and food systems knowledge issues for Iranian school-leavers. Semi-structured face-to-face or telephone interviews were conducted with invited experts between September and Dec 2012. Interviews lasted between 15–120 min.

All interviews were conducted in Farsi by the first author for whom Farsi is her first language. Interviews were recorded using two recording devices to ensure a backup. The first author transcribed the interviews in Farsi and then translated them into English for analysis. Examples of the interview questions are:

- What do you consider the average school-leaver should broadly know about nutrition and food systems? (What do students need to learn before school-leaving age?)
- If we try to outline the most important issues to be included in the education curriculum, what do you think are most important? Why?
- What food-related skills do you think students need to develop?

### Data Analysis

English translations of the interview transcripts were uploaded to NVivo (QSR NVivo 10) for analysis and were subsequently coded. The constant comparative method was used to identify emerging themes (21). In this study, the “step by step” strategy for constant comparative method was used (22. The first three steps of this approach relevant to our study data included: 1) comparison within one interview to develop categories and to label them with suitable codes; 2) comparison between interviews that belong to the identical group of experts who share quite similar experience (e.g. group of nutritionists, group of food scientists); and 3) comparison between interviews from different groups to develop and deepen the information and “complete the picture” (22). The coding was undertaken by two of the authors in an iterative manner and some manual coding also was undertaken as measures to confirm reliability of the coding process.

## Results

### Sample Characteristics

The final list of participants, N=28, included five public health nutritionists, five nutritionists, five dietitians, four food scientists, two environmental scientists, two veterinary physicians, one agriculture scientist and four high school teachers (one health teacher, one home economics’ teacher, one agriculture science teacher and one food science teacher) from Tehran, Fars, Isfahan and Gilan, Iran. They were all experienced in food-related education programs and/or food-related policymaking and/or active in food-related research programs.

There were similarities amongst the findings within the same group of experts (e.g. environmental scientists, food scientists). Interviewing a range of food-related experts provided multiple perspectives on nutrition and food systems knowledge and overcame the potential limitation of interview data reporting selective issues reflecting the views of one or two discipline areas.

Participants believed nutrition and food systems knowledge was necessary for Iranian school-leavers because food-related issues were embedded within people’s daily lives. They highlighted the need for a strategic, coherent, continuous and long-term teaching program from the earliest stages within the formal education system until school-leaving age. Other significant issues raised by almost all the participants was the need to focus on food knowledge issues helpful for “routine life” or information for “everyday use” and “practical skills”, and the importance of avoiding “professional” and “technical” information in education programs.

### Important areas of nutrition and food systems knowledge

**Fig. 1:**
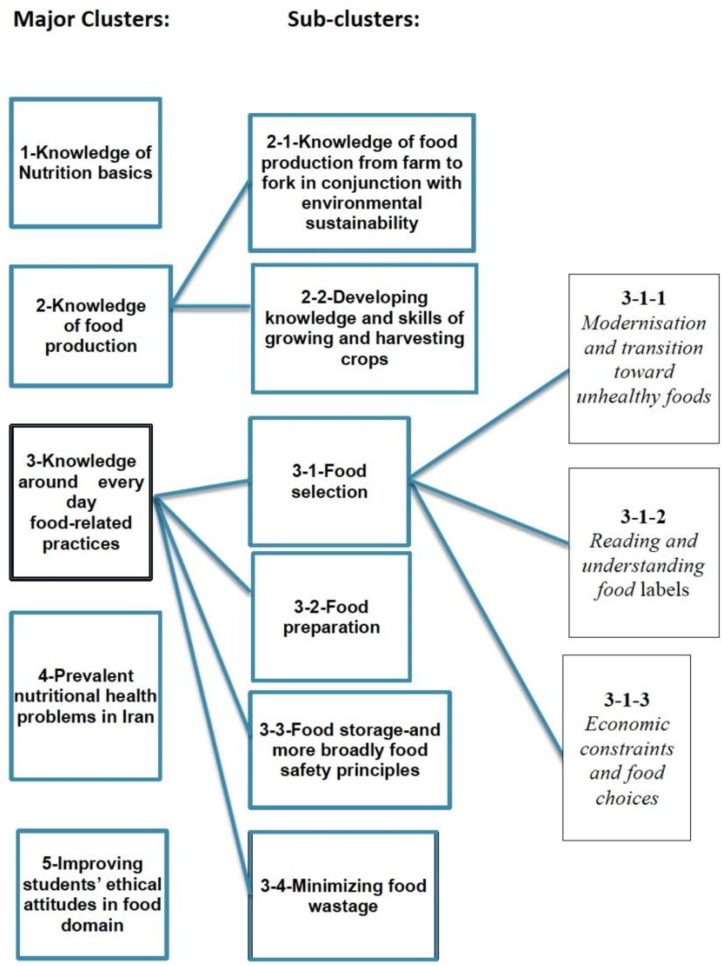
Respondents’ views of important nutrition and food systems knowledge issues for Iranian school-leavers

Five major nutrition and food systems knowledge clusters and several sub-clusters were identified, as depicted in Fig. 1. The major knowledge clusters were nutrition basics, food production, every day food-related practices, prevalent nutrition-related health problems in Iran, and ethical attitudes in the food domain. These clusters are presented in the following sections.

### 1-Knowledge of nutrition basics

This aspect of knowledge, mentioned by most of the participants, included key nutrition recommendations within the national policy guidelines, including the Dietary Guidelines, Food Pyramid and Thrifty Food Basket designed for Iranians.

The major focus was on knowledge of the five food groups; nutritious alternatives within each food group; keeping diversity and balance in consumption of foods from all major food groups, and daily consumption of breakfast. It was identified important to acknowledge and be familiar with the nutrition needs of high-risk population groups, particularly pregnant women, breastfeeding women and children under five, mainly in the context that “future school-leavers might one day become “pregnant” or “a parent”.

### 2-Knowledge of food production

It was important to know “where foods come from” and “how foods are produced and provided”. Major focuses were on: a) food production from farm to fork in conjunction with environmental sustainability and b) knowledge and skills for growing crops

#### 2-1 Food production from farm to fork in conjunction with environmental sustainability

Participants raised the need for awareness of food production procedures in relation to different food groups (e.g. dairy products and cereal foods) from farm to fork. At the same time, they raised the need to avoid “unnecessary”, “theoretical”, “technical” and “professional” information. “For example, school-leavers need to be familiar with milking cows in livestock farm, ways of storing milk, transporting milk to the factory, pasteurising milk, and producing yogurt, cheese, etc., also transferring the products to the market and issues around milk adulteration like adding water or starch to it” (10-R).

Environmental scientists and some public health nutritionists emphasized knowledge of the negative effects on the environment of inappropriate food production systems. Three issues were identified.

Firstly, the significant use of natural resources for producing animal-based proteins compared to plant foods. Secondly, the negative effects of inappropriate agricultural practices on the environment through inappropriate management and use of chemical fertilizers and pesticides. Thirdly, they raised the harmful environmental effects caused by intensive livestock production systems, including increasing greenhouse gas emissions and overuse and damage to natural resources.

“We should highlight the importance of protecting our environment because school-leavers should not only care about what they eat but also about the environment that we are living in - the land, soil, and air. “(8-M)

#### 2-2 Developing knowledge and skills of growing and harvesting crops

A few participants believed students and school leavers, particularly rural students, need some knowledge of growing and harvesting. They asserted that equipping students with this knowledge might positively affect their attitudes, knowledge, and skills regarding gardening and farm-related occupations.

“In Iran, we have proper agricultural lands and it is needed to add agriculture topics within school curriculum … Then they might find enough self-esteem to start farm-related jobs” (11-SH).

### 3-Knowledge around everyday food practices

Each of the study participants mentioned a few knowledge issues related to everyday food practices, including knowledge of food selection, food preparation, food storage (more broadly food safety), and minimizing food wastage.

“It is very important for school-leavers to have some knowledge about food labeling, food storage, and food preparation and cooking” (19-J).

“We haven’t done considerable work on food selection knowledge domain in our country, not for adults, nor for kids. This is a gap in Iran and we need to work on it.” 18-A).

#### 3-1 Food selection

The focuses of knowledge for food selection were on: a) modernization and food transition toward unhealthy foods; b) reading and understanding food labels, and c) economic constraints and food choices

##### 3-1-1 Modernization and food transition toward unhealthy foods

Participants raised the significant role of globalization and modernization in transition toward the consumption of “tasty” and : “convenient” foods. This was particularly important as they expressed the view that shift toward consumption of “junk foods”, “highly processed foods”, “fast food” and “take away” was associated with the rising prevalence of nutrition-related health problems in Iran. Participants believed that education programs should provide regular warnings about the disadvantages of these unhealthy foods.

Some participants were of the view that regular education (in conjunction with feeding children with healthy foods from early childhood in the home setting) was likely to influence later healthy food selection behaviors.

“In Iran, we have had lots of dramatic changes. These changes have been a sort of modernization. Dietary culture has changed. Lots of people mainly eat out of home” (15-GH)

##### 3-1-2 Reading and understanding food labels

Several participants believed that improvements in food labels were required for various food products. They expressed a view that if people’s knowledge were improved, this would increase the demand for healthier products and for the development of more appropriate food labels for all foods. This would lead to positive influences over food manufacturers and other relevant organizations.

##### 3-1-3 Economic constraints and food choices

The financial constraints on some Iranian families were considered to affect their healthy food choices in a negative way. Budget management tips were suggested to be incorporated into education programs. The identified budget tips included: equipping students with knowledge of accessible and affordable alternatives within each food group to provide their nutritional requirements; encouraging students to buy seasonal foods (e.g. for fruits and vegetables); and informing students about equivalent nutritional values of some cheap foods versus similar expensive products (e.g. a cheap small apple is similar to a large expensive apple).

“We always say do not eat fast foods and eat healthy foods. However, some factors are important in this area like economic issues. For example, fast foods, take away foods and some pre-prepared foods are cheap, quick and accessible. Thus in our education and food-related planning, we need to consider these issues” (18-A)

“They need to know what are the best choices based on their economic power” (15-Gh)

#### 3-2 Food preparation and cooking skills

Some participants (particularly teachers) highlighted the need for the acquisition of cooking skills, as students would soon become independent and need to cook for themselves and/or their family members.

Some experts referred to the positive culture of Iranians cooking foods at home. However, they also identified the need to modify some poor practices (e.g. frying vegetables for a long time, using too much oil and consuming too much rice). They believed that the provision of simple, practical tips for healthy cooking would be helpful, for example poaching, steaming and grilling methods versus deep-frying; cooking meat thoroughly for safety reasons, and limiting salt and oil use.

“It is necessary for school-leavers to have some skills of food preparation at home and also healthy methods of traditional cooking” (5-J)

“For example, we can mention that a proper simple method for cooking vegetables to keeps their nutrients is steaming. When you are steaming rice, put the vegetables on top of it” (14-S)

#### 3-3 Knowledge of food storage and food safety principles

Participants from different specialties believed that to reduce the incidences of foodborne illnesses, it was necessary to improve school-leavers’ knowledge of safe food practices during the preparation, cooking, and storage of foods at home. They also noted relevant information specific to the food situation in Iran, including appropriate methods of consuming tinned foods (i.e. boiling tins for 20 min before opening); skills for shopping for safe, non-packed foods like meat and fish; and the need for caution regarding some food products because of overuse of pesticides and other harmful substances in the production of some agricultural products (e.g. phosphate in some vegetables) and animal products (e.g. nitrate in cows’ milk). They considered such information would allow young people to make more informed food choices and enable them to put pressure on producers to meet the food production regulations.

“Iranian school-leavers might be involved in food preparation and storage at home and it is important to increase their knowledge of food safety issues to prevent food-borne illnesses” (4-H)

#### 3-4 Minimization of food wastage

Everyday food practices also included knowledge and skills for minimizing food wastage at the household and production level. For minimizing food wastage in households some experts mentioned the importance of proper storage, planned shopping and selecting food products with environmental friendly packages.

### 4-Knowledge of prevalent nutrition problems in Iran

The dietitians, nutritionists, and public health nutritionists focused on education about common nutrition-related health problems in Iran such as overweight, obesity, diabetes, cardiovascular diseases and micronutrient deficiencies (e.g. iron, zinc, vitamin D and vitamin B). Two participants raised the need for informing students about eating disorders (e.g. anorexia and bulimia nervosa), particularly among teenagers and young adults. The participants assumed that awareness about the consequences of prevalent nutrition-related health problems, in conjunction with the provision of nutrition and physical activity recommendations, would be helpful for the prevention and management of such problems.

“At present, we are struggling with chronic diseases in Iran and nutrition-related awareness is really crucial” (11-SH)

### 5-Improving students’ ethical attitudes in the food domain

A few participants mentioned ethical attitudes of students in the food domain, particularly in terms of animal welfare and environmental sustainability. These experts highlighted animal welfare issues in livestock production systems (e.g. methods of raising farm animals and the benefits of free-range methods). The participants also highlighted the need for providing insights into the value of reducing consumption of animal-based proteins to fulfill daily nutrition requirements, and the importance of respecting and preserving nature through better management techniques and water and soil use. The ethical importance of minimizing food-related wastage was also raised.

“Those who respect nature, creatures, earth, soil and plants and try to preserve the world for the next generations have valuable attitudes ” (11-SH).

### Discussion

This study identified a broad range of nutrition and food systems issues that nutrition and food system experts in Iran considered were important for students to learn during schooling. The results collectively present a broad perspective of nutrition and food systems knowledge issues reflective of the NNS framework and its principles (23). The wide range of nutrition and food systems knowledge issues was not unexpected, as broad, open-ended questions were used in the interviews and the participants were experts from a range of fields relating to nutrition and food systems.

Few previous studies have considered broadly based nutrition and food system knowledge. One comprehensive investigation focused on food literacy and its components, however ethical dimensions within the food domain, such as environmental sustainability and animal welfare, were not considered (24). Of the food knowledge areas identified in this study, three have been consistently reported previously and two less frequently discussed in food and nutrition knowledge literature.

Knowledge of nutrition basics, particularly with reference to national policy guidelines, has been identified in previous studies (25–27). This focus reflects many of the participants’ attitudes toward the important roles of food-based dietary guidelines for Iranian population groups that take into consideration lifestyle changes, socio-economic situations and nutritional transition issues, which have led to increases in the prevalence of chronic diseases and micronutrient deficiencies (28).

The expressed need for ‘knowledge about everyday food-related practices’ is consistent with the findings in other studies. Previous literature has identified reading and understanding food labels (29), developing cooking skills (7), and knowledge of food safety issues (5) as important areas of knowledge. An additional issue raised in this study was the importance of knowledge about nutrition transition issues, including consumption of junk foods, fast foods, highly processed foods and take away foods, and the need for cautioning students about their potential harms. The existence of nutrition transition issues have been reported in the literature since 1970s (30) and reported in Iran (31) but it has not previously been highlighted as an important aspect of knowledge to include in school curricula.

The need for school students to have knowledge of prevalent nutrition-related health problems in Iran, in conjunction with nutrition-related recommendations for prevention and management of chronic diseases, were also reported in this study. This is consistent with previous work which refers to nutrition knowledge as an essential element of health literacy (32).

This study identified two areas of food knowledge, raised less frequently in the food and nutrition knowledge literature. One area was ‘knowledge of food production’ with a focus on environmental sustainability. While research on sustainable food systems has been reported (33–34), only a few studies have explored the knowledge required of food production or of food systems more broadly. One previous study surveyed high school students in the United States to assess their knowledge, attitudes, and experiences of the food system. A high level of detail of food systems content knowledge was explored in that study (9), as it was the main focus of the study. A lesser focus on food system knowledge in the current study may reflect Iranian experts’ lower expectations of students’ awareness of food systems, their preference for only teaching the basics of food production systems or that food system knowledge was being explored within a more broadly based discussion.

In the food production knowledge area, the need for developing skills in growing and harvesting crops was identified. This was mainly aimed at the improvement of students’ skills relating to, and attitudes towards, farm-related jobs. This is different to previous studies, where food production knowledge issues were focused more on gardening education programs to improve dietary behaviors such as fruit and vegetable intakes (35). Enhancement of school leavers’ ethical attitudes in the food domain, particularly in terms of environmental sustainability issues in relation to food production systems and animal welfare, has been reported least frequently in previous food knowledge literature. Consumers, policymakers and producers around the world had become more mindful of farm-animal welfare (36). Other literature discusses the influences of consumers’ food choices or impacts of food production systems on climate change and the environment (37). The current study identified the importance of ethical food-related issues that enable school-leavers to make value-based food choices.

Study findings were analyzed using the multidimensional framework for knowledge and learning (20). The range of areas of knowledge identified by the participants reflected all three facets of knowledge including explicit (e.g. recommendations in policy guides), implicit (e.g. cooking skills and growing and harvesting crops) and emancipatory (animal welfare and environmental sustainability). However, the major foci of the participants’ responses were on knowledge of practical issues, information for everyday use and developing practical skills. Thus by considering Yang’s epistemology of knowledge, overall the participants had greater focus on implicit knowledge (practice) and those specific parts of explicit knowledge (scientific principles) that could directly affect implicit knowledge. Only a few of the participants highlighted the critical role of value-based issues (emancipatory knowledge) in food education programs.

Utilization of Yang’s knowledge framework in this study provides a broader understanding of nutrition and food-related topics in education programs. Adoption of such theoretical frameworks is not common in the food and nutrition knowledge domain. This may be one reason why the multidisciplinary nature of food and nutrition science has not strongly reflected in education programs to date.

## Conclusion

Findings of current study provide a guide to curriculum developers and policy makers to assess current education curricula in order to optimize students’ knowledge of nutrition and food systems.

## Ethical considerations

Ethical issues (Including plagiarism, informed consent, misconduct, data fabrication and/or falsification, double publication and/or submission, redundancy, etc.) have been completely observed by the authors.

## References

[1] Perez-Rodrigo C Aranceta J (2003). Nutrition education in schools: experiences and challenges. Eur J Clin Nutr, 57 (S1): S82–85. 12947462 10.1038/sj.ejcn.1601824

[2] Carraway-Stage V Hovland J Showers C Díaz S Duffrin MW (2015). Food-Based Science Curriculum Yields Gains in Nutrition Knowledge. J Sch Health, 85(4): 231–240. 25731197 10.1111/josh.12243 PMC7489299

[3] Kazemian R Ghasemi H Movahhed T Kazemian A (2014). Health education in primary school textbooks in iran in school year 2010–2011. J Dent (Tehran), 11(5): 536–544. 25628680 PMC4290773

[4] Perez-Rodrigo C Aranceta J (2001). School-based nutrition education: lessons learned and new perspectives. Public Health Nutr, 4(1a): 131–139. 11255503 10.1079/phn2000108

[5] Kennedy J Jackson V Cowan C Blair I McDowell D Bolton D (2005). Consumer food safety knowledge. Br Food J, 107(7):441–452. 10.4315/0362-028x-68.7.1421 16013380

[6] Campos S Doxey J Hammond D (2011). Nutrition labels on pre-packaged foods: a systematic review. Public Health Nutr, 14(8):1496–1506. 21241532 10.1017/S1368980010003290

[7] Hartmann C Dohle S Siegrist M (2013). Importance of cooking skills for balanced food choices. Appetite, 65:125–131. 23402717 10.1016/j.appet.2013.01.016

[8] Wardle J Parmenter K Waller J (2000). Nutrition knowledge and food intake. Appetite, 34(3):269–275. 10888290 10.1006/appe.1999.0311

[9] Harmon AH Maretzki AN (2006). A Survey of Food System Knowledge, Attitudes, and Experiences among High School Students. J Hunger Environ Nutr, 1(1): 59–82.

[10] Lawrence MD Worsley T (2007). Concepts and guiding principles. In: Public health nutrition: from principles to practice. Crows Nest, N.S.W: Allen & Unwin; pp; 5–25.

[11] Ahmadi A Torkamani P Sohrabi Z Ghahremani F (2013). Nutrition knowledge: application and perception of food labels among women. Pak J Biol Sci, 16(24): 2026–2030. 24517023 10.3923/pjbs.2013.2026.2030

[12] Ahadi Z Larijani B Heshmat R (2014). Knowledge, attitude and practice of urban and rural households towards principles of nutrition in Iran: results of NUTRIKAP survey. J Diabetes Metab Disord, 13(1):100. 25551099 10.1186/s40200-014-0100-7 PMC4279888

[13] Askarian M Kabir G Aminbaig M Memish ZA Jafari P (2004). Knowledge, attitudes, and practices of food service staff regarding food hygiene in Shiraz, Iran. Infect Control Hosp Epidemiol, 25(1):16–20. 14756213 10.1086/502285

[14] Amirrood MM Taghdisi MH Shidfar F Gohari MR (2014). The Impact of Training on Women’s Capabilities in Modifying Their Obesity-Related Dietary Behaviors: Applying Family-Centered Empowerment Model. J Res Health Sci. 14(1):75–80. 24402855

[15] Ansari H Farajzadegan Z Hajigholami A Paknahad Z (2014). A randomized field trial for the primary prevention of osteoporosis among adolescent females: Comparison of two methods, mother centered and daughter centered. J Res Med Sci, (8):746–752. 25422660 PMC4235095

[16] Tavassoli E Reisi M Javadzade SH Gharlipour Z Gilasi HR Ashrafi Hafez A (2015). The effect of education on improvement of intake of fruits and vegetables aiming at preventing cardiovascular diseases. Med J Islam Repub Iran, 29: 183. 26034736 PMC4431428

[17] Fallah F Pourabbas A Delpisheh A Veisani Y Shadnoush M (2013). Effects of nutrition education on levels of nutritional awareness of pregnant women in Western Iran. Int J Endocrinol Metab, 11(3):175–178. 24348589 10.5812/ijem.9122 PMC3860105

[18] Mirmiran P Azadbakht L Azizi F (2007). Dietary behaviour of Tehranian adolescents does not accord with their nutritional knowledge. Public Health Nutr, 10(9):897–901. 17517151 10.1017/S1368980007246701

[19] Azizi M Rahmani-Nia F Malaee M Malaee M Khosravi N (2010). Study of nutritional knowledge and attitudes of elite college athletes in Iran. Braz J Biomotricity, 4(2):105–112.

[20] Yang B (2003). Toward a Holistic Theory of Knowledge and Adult Learning. Hum. Resource Dev. Rev, 2(2):106–129.

[21] Charmaz K (2006). Constructing Grounded Theory : A Practical Guide Through Qualitative Analysis / Kathy Charmaz [e-book]. London : SAGE. Available from: UOW Catalogue, Ipswich, MA.

[22] Boeije H (2002). A Purposeful Approach to the Constant Comparative Method in the Analysis of Qualitative Interviews. Qual Quant, 36(4): 391–409.

[23] Beauman C Lötsch B Margetts BM McMichael AJ Meyer-Abich K Oltersdorf U (2005). The principles, definition and dimensions of the new nutrition science. Public Health Nutr, 8(6a):695–198. 16236202 10.1079/phn2005820

[24] Vidgen HA Gallegos D (2014). Defining food literacy and its components. Appetite, 76:50–59. 24462490 10.1016/j.appet.2014.01.010

[25] Croll JK Neumark-Sztainer D Story M (2001). Healthy eating: what does it mean to adolescents? J Nutr Educ, 33(4):193–198. 11953240 10.1016/s1499-4046(06)60031-6

[26] Kandiah J Jones C Ignico A (2005). Nutrition Knowledge and Food Choices of Elementary School Children. Early Child Dev Care, 41(2):40–43.

[27] Kothe EJ Mullan BA (2011). Perceptions of fruit and vegetable dietary guidelines among Australian young adults. Nutr Diet, 68(4):262–266.

[28] Safavi SM Omidvar N Djazayery A Minaie M Hooshiarrad A Sheikoleslam R (2007). Development of Food-Based Dietary Guidelines for Iran: A Preliminary Report. Ann Nutr Metab, 51(2):32–35.

[29] Sharf M Sela R Zentner G Shoob H Shai I Stein-Zamir C (2012). Figuring out food labels. Young adults’ understanding of nutritional information presented on food labels is inadequate. Appetite, 58(2): 531–534. 22210347 10.1016/j.appet.2011.12.010

[30] Popkin BM Adair LS Ng SW (2012). Global nutrition transition and the pandemic of obesity in developing countries. Nutr Rev, 2012;70(1):3–21. 22221213 10.1111/j.1753-4887.2011.00456.x PMC3257829

[31] Ghassemi H Harrison G Mohammad K (2002). An accelerated nutrition transition in Iran. Public Health Nutr, 5(1a):149–155. 12027278 10.1079/PHN2001287

[32] Spronk I Kullen C Burdon C O’Connor H (2014). Relationship between nutrition knowledge and dietary intake. Br J Nutr, 111(10):1713. 24621991 10.1017/S0007114514000087

[33] Renting H Marsden TK Banks J (2003). Understanding alternative food networks: exploring the role of short food supply chains in rural development. Environ Plan A, 35(3):393.

[34] McCarthy E Wolff C Bianco-Simeral S Crozier J Goto K (2012). The Effects of a School-Based Nutrition Intervention on Fruit and Vegetable Preferences, Self-Efficacy, and Consumption Among Low-Income, Hispanic and White Middle-School Students. J Child Nutr Manag, 36(2).

[35] Lautenschlager L Smith C (2007). Understanding gardening and dietary habits among youth garden program participants using the Theory of Planned Behavior. Appetite, 49(1):122–130. 17336424 10.1016/j.appet.2007.01.002

[36] Heng Y Peterson HH Li X (2013). Consumer attitudes toward farm-animal welfare: the case of laying hens. J Agr Resour Econ, 38(3):418–434.

[37] Carlsson-Kanyama A (1998). Climate change and dietary choices — how can emissions of greenhouse gases from food consumption be reduced? Food Policy, 23(3–4):277–293.

